# IMMERSIVE VIRTUAL REALITY INTERVENTIONS FOR OLDER ADULTS: A SERIES OF META-ANALYSES

**DOI:** 10.1093/geroni/igad104.2818

**Published:** 2023-12-21

**Authors:** Allison Walden, Christopher Griffith, Payton Downey, Leilani Feliciano

**Affiliations:** University of Colorado Colorado Springs, Colorado Springs, Colorado, United States; University of Colorado at Colorado Springs, Colorado Springs, Colorado, United States; University of Colorado at Colorado Springs, Colorado Springs, Colorado, United States; University of Colorado Colorado Springs, Colorado Springs, Colorado, United States

## Abstract

Many older adults desire to age in place, a term used to describe living independently versus transitioning to a residential care facility. Virtual reality (VR) has the potential to positively impact the functioning of older adults as an assessment or intervention to help compensate for lost or declining functions. VR captures a wide variety of technologies ranging from non-immersive to fully immersive, designed to give the user a feeling of presence. To date, VR interventions have been used with older adults including improving health outcomes, improving functional abilities, and for neuropsychological purposes. A handful of meta-analyses (M-As) attempt to summarize the overall effectiveness of VR interventions with older adults, but many contain methodological issues and cover the gamut of VR technology. The present M-A fills a gap in the literature by assessing the literature on immersive VR interventions for older adults. Sixteen articles met inclusion for meta-analysis, and 41 effect sizes (ES) were calculated. ES were grouped by category, neuropsychological, health/mental health, functional, and eight meta-analyses were performed with one exploratory analysis. Results revealed small, significant ES for VR interventions on measures of global cognition, simple attention tasks, executive functioning tasks, anxiety ratings, and pain ratings. Nonsignificant ES were found for stress ratings, gait speed, depression ratings, and TUG task (exploratory M-A). Continuing to develop affordable, safe interventions to assist with aging in place is important as the number of older adults continues to increase. Immersive VR is a promising avenue to continue exploring to better assist the aging population.

